# Targeted imaging and induction of apoptosis of drug-resistant hepatoma cells by miR-122-loaded graphene-InP nanocompounds

**DOI:** 10.1186/s12951-016-0237-2

**Published:** 2017-01-23

**Authors:** Xin Zeng, Yi Yuan, Ting Wang, Han Wang, Xianyun Hu, Ziyi Fu, Gen Zhang, Bin Liu, Guangming Lu

**Affiliations:** 1 Department of Medical Imaging, Jingling Hospital, School of Medicine, Nanjing Universtiy, Nanjing, 210002 China; 2 Nanjing Maternity and Child Health Medical Institute, Obstetrics and Gynecology Hospital Affiliated to Nanjing Medical University, Nanjing, 210004 China; 3 Institute of Stomatology, Nanjing Medical University, Nanjing, 210029 China; 4 Department of Cell Biology, School of Basic Medical Sciences, Nanjing Medical University, Nanjing, 210029 China; 5 Department of Biochemistry, Qiannan Medical College for Nationalities, Duyun, 558000 China; 6 Department of Biomedical Engineering, School of Basic Medical Sciences, Nanjing Medical University, Nanjing, 210029 China

**Keywords:** Graphene oxide, Quantum dots, MiR-122, Cell apoptosis, Near infrared, Liver cancer

## Abstract

**Background:**

Currently, graphene oxide has attracted growing attention as a drug delivery system due to its unique characteristics. Furthermore, utilization of microRNAs as biomarkers and therapeutic strategies would be particularly attractive because of their biological mechanisms and relatively low toxicity. Therefore, we have developed functionalized nanocompounds consisted of graphene oxide, quantum dots and microRNA, which induced cancer cells apoptosis along with targeted imaging.

**Results:**

In the present study, we synthesized a kind of graphene-P-gp loaded with miR-122-InP@ZnS quantum dots nanocomposites (GPMQNs) that, in the presence of glutathione, provides controlled release of miR-122. The miR-122 actively targeted liver tumor cells and induced their apoptosis, including drug-resistant liver tumor cells. We also explored the near-infrared fluorescence and potential utility for targeting imaging of InP@ZnS quantum dots. To further understand the molecular mechanism of GPMQNs-induced apoptosis of drug-resistant HepG2/ADM hepatoma cells, the relevant apoptosis proteins and signal pathways were explored in vitro and in vivo. Furthermore, near-infrared GPMQNs, which exhibited reduced photon scattering and auto-fluorescence, were applied for tumor imaging in vivo to allow for deep tissue penetration and three-dimensional imaging.

**Conclusion:**

In conclusion, techniques using GPMQNs could provide a novel targeted treatment for liver cancer, which possessed properties of targeted imaging, low toxicity, and controlled release.

**Electronic supplementary material:**

The online version of this article (doi:10.1186/s12951-016-0237-2) contains supplementary material, which is available to authorized users.

## Background

Liver cancer is the third-leading cause of cancer-related death in the world. The high-incidence regions include sub-Saharan Africa, the People’s Republic of China, Hong Kong, and Taiwan [1]. Chemotherapy is an important treatment for liver cancer, but cancer cells can acquire resistance to a wide variety of unrelated drugs when they are exposed to a chemotherapeutic agent. This phenomenon is termed multi-drug resistance (MDR). The molecular basis of a major form of MDR is the overexpression of P-glycoprotein (P-gp) and the increased activity of glutathione transferase (GST) [2–4]. Overcoming drug resistance to chemotherapy has become one of the primary goals of modern approaches to cancer therapy.

The application of nanotechnology in cancer treatment offers some exciting possibilities, including the possibility of destroying cancer tumors with minimal damage to healthy tissues and organs, as well as the detection and elimination of developing tumors [5, 6]. Recently, liposomes, metal nanocarriers and polymers have been investigated for their potential multifunctional uses as therapeutic agents, delivery vehicles and imaging agents capable of being visualized by magnetic resonance imaging (MRI) or optical imaging techniques [7, 8]. The surfaces of these nanocarriers are typically conjugated with a targeting molecule such as a specific antibody (e.g., an antibody against P-gp on the surface of drug resistant tumor cells) [9]. The key issues in this process include the design and synthesis of nanocarriers, the choice of targeting molecules, the assembly of drug nanocarriers and targeting molecules for the integration of diagnosis and treatment [10].

As an important biomarker and imaging nano-optical probe, quantum dots (QDs) play an important role in cell labeling and in vivo imaging [11, 12]. However, because traditional QDs contain lead, cadmium, mercury or other high toxic heavy metals, the use of fluorescent QDs in vitro and in vivo has been restricted. Near-infrared fluorescence imaging within the wavelength range of 650–950 nm offers several advantages for tumor and in vivo imaging owing to its low absorption and auto-fluorescence from organisms and tissues in the near-infrared spectral range, which can minimize background interference, improve tissue depth penetration, image sensitivity and function noninvasively [13, 14]. Indium phosphide (core)-zinc sulfide (shell) InP@ZnS core–shell nanocomposites exhibit a very large stock shift, which leads to the appearance of near-infrared region fluorescent emission. With high quality, low toxicity and bright luminescence, InP@ZnS QDs have been used in diagnostic near-infrared imaging for the early detection of cancer [15, 16]. Herein, we report the physicochemical characteristics and bioapplications of novel hybrid nanocomposites of graphene oxide and InP@ZnS QDs that are bound to biomolecules (P-gp antibody) for multimodal targeting and treatment of drug-resistant cancer.

With the development of biotechnology, microRNAs (miRNAs) have been considered as important biomarkers since abnormal expression of specific miRNAs is associated with many diseases such as cancer, and the understanding of a variety of miRNA regulatory pathways in liver cancer has been gradually growing [17]. In multiple expression research of miRNAs profile, the expression of miR-122 in many hepatoma cells lines was found to be down-regulated. As a hepatic-abundant miRNA, miR-122 is involved in the regulation of cancer cell migration and chemoresistance in liver. And with increased miR-122 expression, the intrahepatic metastasis of liver cancer is significantly reduced or absent. In hepatoma tissues, the cyclin G1/tumor suppressor gene p53, apoptosis inhibitor gene Bcl-W and other related genes are targets of miR-122 [18, 19]. Based on its biological mechanisms and relatively low toxicity, miR-122 was chosen to control drug-resistant hepatocellular carcinoma cell growth and apoptosis in our study.

In our previous work, we exploited the possibility of combining the properties of gold nanoclusters and reduced graphene oxide (RGO) to design nanocomposites suitable for drug delivery to and imaging of cancer cells [20]. In the following study, we intended to develop a combination of monoclonal P-glycoprotein (P-gp) antibodies and miR-122 loaded on graphene oxide InP@ZnS QDs (GPMQNs), which should promote drug-resistant tumor cell apoptosis and exhibit targeted controlled-release properties. Due to the function of the P-gp antibody, the GPMQNs could provide targeted drug delivery. On the other hand, combining with glutathione (GSH) could displace miR-122, which helped to control drug release.

GPMQNs were used to induce the apoptosis of drug-resistant human HepG2/ADM hepatoma cells. Meanwhile, apoptosis-related proteins and the apoptosis signaling pathway were investigated. And the ability of the GPMQNs to provide near-infrared imaging of HepG2/ADM tumors was also explored. In conclusion, the present study could provide innovative therapeutic approaches for cancer treatments with following advantages. (1) Inhibition of tumor growth and induction of tumor cell apoptosis by GPMQNs were demonstrated in vitro and in vivo, with the characteristics of high selectivity and specificity toward target cancer cells with low cytotoxicity and controlled release. (2) miR-122 was selected instead of chemical drugs due to its higher safety and avoidance of MDR for chemotherapy. (3) Photothermal therapy to kill cancer cells could be applied by exciting GPMQNs with a semiconductor laser, forming a kind of combination therapy.

## Methods

### GPMQNs nanocomposites characterization

Sodium chloride (NaCl), sulfuric acid (H_2_SO_4_), potassium permanganate (KMnO_4_), hydrochloric acid (HCl), sodium hydroxide (NaOH), chloroacetic acid, 1-(3-dimethylaminopropyl)-3-ethylcarbodiimide (EDC), tris(trimethylsilyl)phosphine (P(TMS)_3_, 1-octylamine, 1-octadecene (ODE), indium acetate (In(AC)_3_), myristic acid (MA), and amine were purchased from Shanghai Chemical Reagent Co. Ltd (China). RPMI-1640 medium and fetal calf serum (FCS) were purchased from Thermo Fisher Scientific (USA). Penicillin, streptomycin, adriamycin, GSH, acridine orange/ethidium bromide (AO/EB), 3-(4,5-Dimethylthiazol-2-yl)-2,5-diphenyltetrazolium bromide (MTT), dimethyl sulfoxide (DMSO), hematoxylin-eosin (HE) were purchased from Sigma-Aldrich (USA). MiR-122 was synthesized by Shanghai Sangon Biologic Engineering Technology and Service Co. Ltd. (China).

Graphene oxide was self-made. The synthesis process of graphene oxide loaded with P-gp antibody (Sigma-Aldrich, USA) was described as following. 1 g graphite and 50 g NaCl were milled for 10 min and dissolved in water. The ground material was stirred for 8 h with 98% H_2_SO_4_ (23 mL). Next, 3 g KMnO_4_ was gradually added to the mixture, and the reaction temperature was maintained below 20 °C. Then, the solution was mixed at 38 °C for 30 min and stirred at 70 °C for 45 min, and H_2_O (46 mL) was added, after which the mixture was maintained at 98 °C for 30 min prior to the gradual addition of 30% H_2_O_2_ (10 mL). After filtration and recovery, 5% HCl was used to dissolve the filter material, which was then further dissolved in double-distilled H_2_O (ddH_2_O). The graphene oxide (1 mg, 1.5 mg mL^−1^) of different sizes was separated using gradient centrifugation at the following centrifugal conditions: 10,000 rpm 2 h, 20,000 rpm 2 h, 30,000 rpm 2 h, 40,000 rpm 2 h, and 50,000 rpm 2 h. Then, the centrifuged nanocompounds were vacuum dried at room temperature. Transmission electron microscope (TEM, JEM2100, JEOL, JPN) was used to observe the samples morphology. Graphene oxide (5 mL, 2 mg mL^−1^) of 300 nm diameter was treated with ultrasound for 1 h. 1.2 g NaOH and 1 g chloroacetic acid (Cl–CH_2_–COOH) were added into the solution, which was then treated with ultrasound for 2 h to form carboxylic acids on the grapheme oxide. To cross-link P-gp antibodies onto the graphene oxide, the P-gp antibody was quantitatively added into the graphene oxide solution, and an EDC catalytic reaction was performed at room temperature, followed by vacuum drying and enhanced chemiluminescence (ECL, GE Healthcare Life Sciences, USA) detection.

InP@ZnS QD synthesis process was described as following. The injection solution was prepared using 0.2 mM P(TMS)_3_ (Alf. 95%) and 2.4 mM 1-octylamine (Alf. 99%) dissolved in ODE (1.5 mL in total) in a glove box. In a typical synthesis, 0.4 mM In(AC)_3_ (Alf. 99.99%), 1.54 mM MA (Alf. 98%) and 4 g ODE were loaded into a three-neck flask in the total volume of 1 L. The mixture was heated to 188 °C under argon flow, and then the P(TMS)_3_/amine solution prepared in the glove box was injected into the hot reaction mixture. The cold injection solution brought the reaction temperature down to 178 °C for InP nanocrystals growth. To monitor the nanocrystals growth, aliquots were taken at different reaction times for absorption measurements [21].

Preparation and characterization of the GPMQNs system: 1 mg P-gp antibody-modified graphene oxide was added to 1 mL chitosan solution (0.5 mg mL^−1^, 1% HAc, pH 5) and was treated with ultrasound for 1 h. The final product and 0.5 mL (0.001 mg mL^−1^) miR-122-InP@ZnS QDs were mixed and then stirred overnight. The end product of the GPMQNs reaction was vacuum dried. TEM was used to characterize the morphology of the GPMQNs. The same amount of miR-122 and GPMQNs were analyzed using agarose gel electrophoresis in the miR-122 GSH-release experiments with added GSH. The fluorescence emission spectrum of GPMQN was recorded using a fibre optic charge coupled device (CCD) spectrometer (USB4000, Ocean Optics Inc., USA). For the absolute quantum yield measurement, a spec-trometer incorporating an integrating sphere was used (C9920-02, Hamamatsu, JPN). Mean sizes analyses for GPMQN were evaluated by dynamic light-scattering using Zetasizer (size range: 1 nm¨1 mm, Malvern Instruments Ltd., UK), a photo-correlation spectroscopy apparatus.

Nucleic acid release assay: the same amounts of miR-122 of the GPMQN were loaded into the wells of an agarose gel to perform electrophoresis to detect GSH for miR-122 in the sustained-release experiment. For the well without GSH added, no nucleic acid showed bands.

### GPMQNs uptake analysis with near-infrared imaging in vitro

HepG2 cells were maintained in RPMI-1640 medium containing 10% FCS, 100 U mL^−1^ of penicillin, and 100 μg mL^−1^ of streptomycin at 37 °C with 5% CO_2_. To develop the drug resistant cell line (HepG2/ADM), adriamycin was added to HepG2 cells in stepwise increasing concentrations, from 0.05 to 2 µg mL^−1^ over 8 months. HepG2/ADM cells were cultured in 6-well plates, and then were treated with GPMQNs for 1 h. After the washing of the cells, confocal fluorescence microscopy (excitation wavelength at 600 nm) was used to observe the intracellular near-infrared fluorescence (CarlZeiss LSM710, Carl Zeiss, German), and small animal imaging experiments were used to observe the intracellular near-infrared fluorescence.

HepG2/ADM cells were seeded in a 96-well plate (2 × 10^3^ cells/well). After an overnight culture, the cells were treated with miR-122 in Lipofectamine-2000, GPMQNs without miR-122, or GPMQNs. The effect of GPMQNs on cancer cell membrane permeability was determined using a lactate dehydrogenase (LDH, Thermo Fisher Scientific, USA) cytotoxicity assay.

### Apoptosis induced by GPMQNs treatment in vitro

HepG2/ADM cells were cultured in 6-well plates and then treated with 10 mg L^−1^ GPMQNs for 24 h. After the washing, the morphology of the HepG2/ADM cells was observed using confocal fluorescence microscopy experiments (excitation wavelength at 600 nm).

HepG2/ADM cells treated with 10 mg L^−1^ GPMQNs were stained with an AO/EB dye mixture and viewed under a fluorescence microscope.

HepG2/ADM cells were plated in 96-well plates (2 × 10^3^ cells/well). After overnight incubation, the cells were treated with various concentrations of GPMQNs. After 36 h, a 20 μL MTT solution (5 mg/mL) aliquot was added into each well. After 4 h of incubation, the supernatant was removed, and 100 μL DMSO was added to each well. The samples were then shaken for 15 min before the optical density was measured at a wavelength of 540 nm. All experiments were performed in triplicate. The relative inhibition of cell growth was expressed as follows:

$$ {\text{Cell}}\;{\text{viability}}\;{{\% }}\;{ = }\;\left( {\left[ {\text{OD}} \right]{\text{test/}}\left[ {\text{OD}} \right]{\text{control}}} \right)\;{{ \times }}\; 1 0 0 {\text{\%}}. $$

GPMQNs used for HepG2/ADM in vitro laser hyperthermia (SLIM-532, Oxxius, France): the laser irradiation experiment involved choosing different wavelengths of semiconductor lasers. HepG2/ADM cells were added to the GPMQNs solution, exposed to a power density of 20 W/cm^−2^ of the semiconductor laser light source and irradiated for 1 min prior to trypan blue staining.

Detection of HepG2/ADM cell DNA fragmentation and apoptosis by flow cytometry (BD Accuri C6, BD, USA): HepG2/ADM cells were treated with miR-122-Lipofectamine 2000, GPMQNs without miR-122, or GPMQNs treatment. The apoptosis rate was evaluated using flow cytometry. Apoptotic DNA in the HepG2/ADM cells was explored using an Apoptotic DNA Ladder Isolation Kit (Biovision, USA) and then separated by agarose gel electrophoresis.

The mechanism of HepG2/ADM cell apoptosis was explored using Western blotting. HepG2/ADM cells were treated with miR-122-Lipofectamine 2000, GPMQNs without miR-122, or GPMQNs for 72 h, and the total proteins were extracted using radioimmunoprecipitation assay (RIPA) buffer (Thermo Fisher Scientific, USA). The protein concentration was determined using a BCA kit (Bio-Rad, USA). Proteins (15 μg) were separated by SDS-PAGE and transferred to nitrocellulose membranes (GE Healthcare Life Sciences, USA). The membranes were blocked in 5% non-fat dry milk followed by incubation with primary antibodies. Then, the membranes were incubated with goat anti-rabbit IgG horseradish peroxidase (HRP)-conjugated secondary antibodies (1:2000, 7074, Cell Signaling Technology (CST), Inc., USA) and developed using ECL (GE Healthcare Life Sciences, USA). To study the related signal transduction pathways, antibodies were used to detect the activated forms of caspase 8 (death receptor pathway), caspase 9 (mitochondrial pathway), caspases 7, 3, 1, proteolytic cleavage of poly-(ADP-ribose) polymerase (PARP), Bcl-2, and Bcl-w, with glyceraldehyde-3-phosphate dehydrogenase (GAPDH) expression level as a control. The primary antibodies used were as follows: anti-caspase 8 (1:1000; 9496, CST, USA), anti-caspase 9 (1:1000; 9502, CST, USA), anti-caspases 7 (1:1000; 8438, CST, USA), anti-caspases 3 (1:1000; 9654, CST, USA), anti-caspases 1 (1:1000; 4199, CST, USA), anti-PARP (1:1000; 5625, CST, USA), anti-Bcl-2 (1:1000; 2872, CST, USA), anti-Bcl-w (1:1000; 2724, CST, USA), anti-GAPDH (1:1000; 2118, CST, USA) rabbit monoclonal antibody.

### GPMQNs treatment to HepG2/ADM tumor-bearing mice for near-infrared imaging in vivo

Mice used in this study were housed in the mouse facility of Model Animal Research Center, Nanjing Medical University, in accordance with Institutional Animal Care and Use Committee (IACUC) approved protocol.

Establishment of the drug-resistant HepG2/ADM nude mice tumor model: HepG2/ADM cells in the logarithmic growth phase were injected into nude mice, and the animals were divided into four groups, each group with 5 mice: (1) normal saline, (2) GPMQNs without miR-122, (3) miR-122-Lipofectamine 2000, and (4) GPMQNs. The tumor cells were inoculated a week later; when the tumor grew to approximately 50 mm^3^ in size, the four groups of nude mice received tail vein injections of the various treatments at 0, 2, 4, 6, 8, 10, 12, 14, 16, and 18 days. On the twentieth day, the tumor was removed and formalin fixed; the size of the tumor was calculated using the formula ${\text{V }}\;{ = }\;{{\pi /6}}\;{{ \times }}\;\left[ {\left( {{\text{A }}\;{ + }\;{\text{B}}} \right) / 2} \right]^{ 3} ,$ where A represents the maximum tumor diameter and B represents the minimum diameter of the tumor.

The animals were anesthetized intraperitoneally and were placed on the table in a side position so that the detector was positioned on the tumor region of the animal. Small animal in vivo imaging was performed using Lumina XR instruments with excitation wavelength at 600 nm (Caliper Life Science, Inc., USA).

### Establishment of tumor cell model in vivo for HepG2/ADM cell apoptosis analysis

In vivo cell apoptosis analysis: histology of tumor tissue from experimental nude mice. Tumor tissue sections were embedded in paraffin wax, and HE staining was performed for detection of cell apoptosis. Five mice from each group were sacrificed at 5 weeks to obtain mice organs (bone, skin, muscle, intestine, liver and tumor). Tissues were digested to measure In and Zn levels. All organs were washed with distilled deionized water and dried on paper towels. The samples were dried to constant weight at 105 °C. The organs were then ground in an agate mortar and digested in aqua regia. After appropriate dilution with ddH_2_O, the metal concentrations in the samples were determined by atomic absorption spectrophotometry.

### Statistical analysis

Results were presented as mean ± standard deviation (SD). A *t* test was performed in each group for each time point. A value of P < 0.05 was considered statistically significant.

## Results

### Synthesis and identification of GPMQNs

**Fig. 1:**
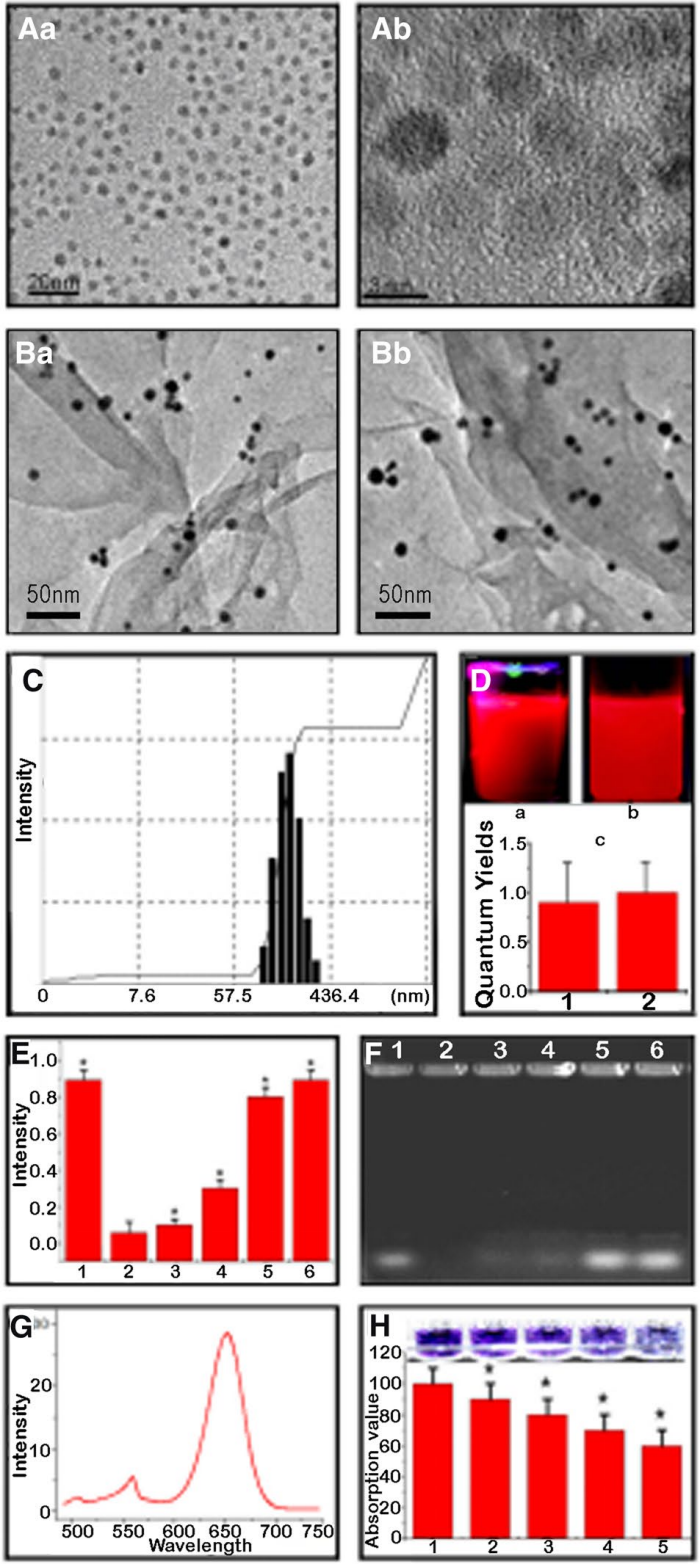
Synthesis and characterization of miR-122-InP QDs-loaded graphene oxide composites. **A** *a* Low magnification image of InP QDs (*Scale bar* 20 nm). **A** *b* HRTEM image of InP QDs (*Scale bar* 3 nm). **B** *a* TEM image of miR-122-InP QDs-loaded graphene oxide composites (*Scale bar* 50 nm). **B** *b* TEM image of GPMQN (*Scale bar* 50 nm). **C** Size distribution of GPMQN in the culture medium characterized by dynamic light scattering. **D** Calculating quantum yields of GPMQNs (*a*) and compare with bare QDs (*b*); (*c*) Histograms of quantum yields of GPMQNs (*a*) and compare with bare QDs (*b*). **E** Verified function of miR-122 by GSH through AO fluorescence assay; *1* AO + miR-122, *2* AO + GPMQN, *3* AO + GPMQNs + 0.2 mM GSH, *4* AO + GPMQNs + 1 mM GSH, *5* AO + GPMQN + 5 mM GSH, *6* AO + GPMQN + 10 mM GSH. **F** Confirmed function of miR-122 release by GSH through agarose gel electrophoresis assay; *1* AO + miR-122, *2* AO + GPMQN, *3* AO + GPMQN + 0.2 mM GSH, *4* AO + GPMQN + 1 mM GSH, *5* AO + GPMQN + 5 mM GSH, *6* AO + GPMQN + 10 mM GSH. **G** Emission spectrum of GPMQN, excitation wavelength at 650 nm. **H** Quantification of P-gp antibody remaining in solution; *1* 0 h, *2* 1 h, *3* 4 h, *4* 8 h, *5* 12 h exposure to graphene oxide (*P < 0.05 compared to the control group)

InP QDs loaded with miR-122 were synthesized and identified by TEM imaging. The average size of the InP QDs was approximately 3 nm (Fig. 1Aa, Ab). However, we found that the InP QDs and miR-122 complexes were approximately 20 nm (Fig. 1Ba). Thus, we speculated that an abundant amount of miR-122 could be loaded onto the InP QDs. As shown in Fig. 1Bb, the GPMQNs nanocomposites (300 nm) were synthesized and characterized. The GPMQNs were also characterized by dynamic light scattering, which measured the hydrodynamic diameter of the nanocomposites in their dispersion state. The mean size of GPMQNs measured in the culture medium was about 300 nm (Fig. 1C). The TEM image indicated a homogeneous distribution of InP QDs on the P-gp antibody-graphene oxide surface with chitosan functionalization. To quantify fluorescence yield of QDs reduced by graphene, we have performed fluorescence yield assessment. We find quantum yields of InP in GPMQNs was not reduced due to the InP fluorescence was near-infrared fluorescence (Fig. 1D). As expected, a small amount of miR-122 of the same size as pure miR-122 (Fig. 1F, lane 1) was released when the concentration of GSH reached 2 mM (Fig. 1F, lane 4). The mobility of miR-122 recovered completely when the final GSH concentration reached 10 mM (Fig. 1F, lane 5). We demonstrated that the InP QDs completely prevented miR-122 from moving to the positive electrode (Fig. 1F, lane 2). The positively charged InP QDs may have counteracted the negative charges of miR-122. However, negatively charged GSH containing a thiol has stronger affinity to InP QDs and the addition of GSH was demonstrated to potentially counteract the positive charge of the InP QDs to some extent by ligand exchange, resulting in the release of miR-122 from the InP QDs. As shown in Fig. 1, the release of miR-122 from the InP QDs was quantified using a nucleic acid release assay, and the results were consistent with the electrophoresis experiment (Fig. 1E). The typical near-infrared fluorescence spectrum of the GPMQNs was approximately 650 nm, as shown in Fig. 1G. Moreover, we also illustrated that the P-gp antibody could be effectively absorbed by graphene oxide (Fig. 1H). The results suggested that P-gp antibody-graphene oxide and GSH might play a critical role in combining miR-122 with GPMQNs to enhance the targeting of miR-122 to cancer cells. The relevant miR-122 loading efficiency was further determined by OD analysis, which indicated that the miR-122 loading onto the GPMQNs was approximately 10%.

### Near-infrared cellular GPMQNs image analysis and intracellular miR-122 accumulation assay

**Fig. 2:**
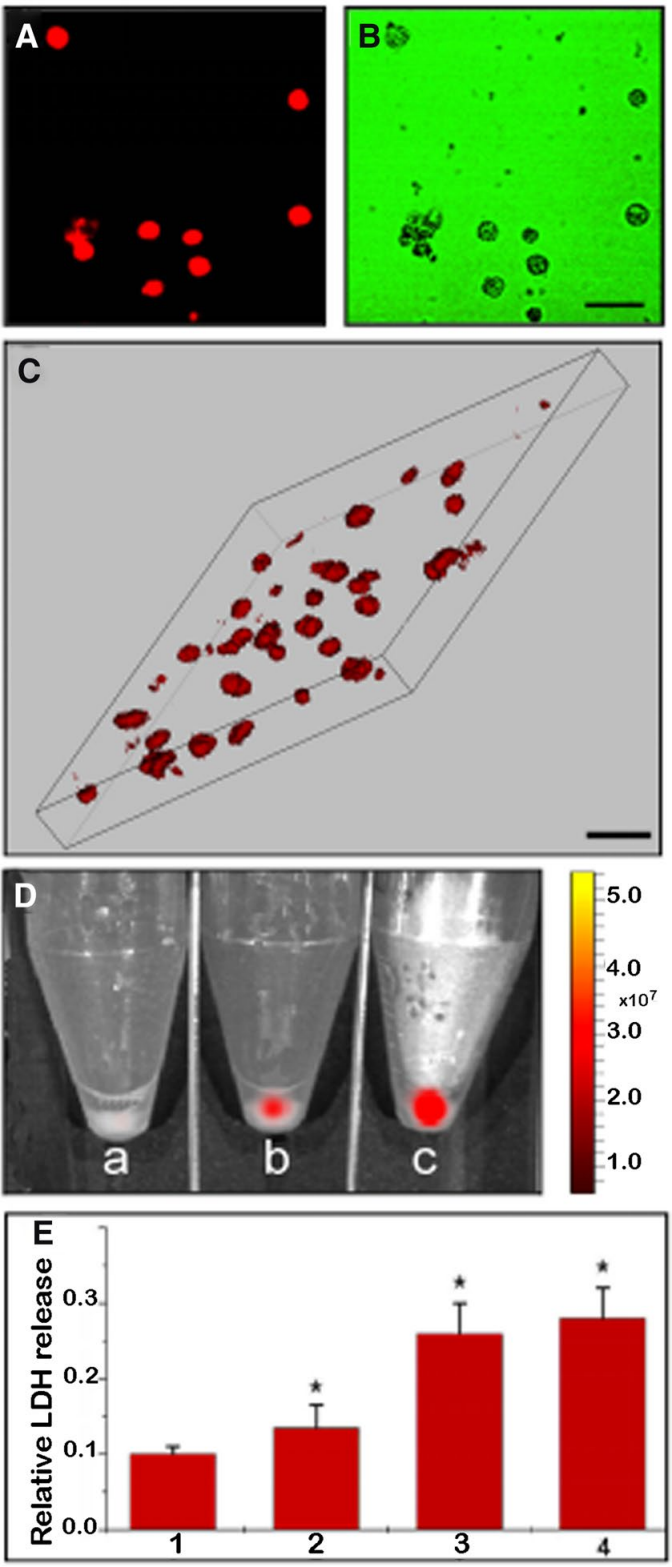
**A** Cellular near-infrared fluorescence and GPMQNs uptake. Inverted fluorescence microscopy of HepG2/ADM cells with 10 mg L^−1^ GPMQNs, **B** Control (*Scale bar 50* *μm*). **B** 3D reconstruction of HepG2/ADM cells treated with 10 mg L^−1^. GPMQNs of near-infrared fluorescence for intracellular distribution (*Scale bar* 50 μm). **D** Whole body optical imaging examination of HepG2/ADM cells incubated with identical 10 mg L^−1^ GPMQNs solutions after 24 h incubation; *a* Control, *b* 1 mg L^−1^ *red fluorescent* modified miR-122, *c* 10 mg L^−1^ GPMQNs containing the *red fluorescent* modified miR-122. **E** Quantitative assay of GPMQNs on cell membrane permeability based on the LDH release assay; *1* untreated control, *2* resistant HepG2/ADM cells transfected with miR-122 (1 mg L^−1^, same concentration as loaded on GPMQN), *3* resistant HepG2/ADM cells incubation with 10 mg L^−1^ GPMQNs without miR-122, *4* resistant HepG2/ADM cells incubation with 10 mg L^−1^ GPMQNs (*P < 0.05 compared to the control group)

Based on the above research, the near-infrared bio-imaging of GPMQNs in HepG2/ADM cell lines was performed using inverted fluorescence microscopy. The near-infrared intracellular fluorescence of HepG2/ADM cells treated with GPMQNs was detected (Fig. 2A, B). The three dimensional (3D) reconstruction of HepG2/ADM cells treated with GPMQNs demonstrated higher intracellular near-infrared GPMQNs distribution (Fig. 2C).

The intracellular fluorescence in HepG2/ADM cells increased dramatically upon treatment with GPMQNs containing red fluorescence-modified miR-122 (Fig. 2Dc), compared with the transfected miR-122 group (Fig. 2Db). The results illustrated that the intracellular miR-122 content was increased after treatment with GPMQNs.

GPMQNs also exhibited an increased impact on cell permeability, as compared with the miR-122 transfection and GPMQNs without miR-122 groups (Fig. 2E).

### Apoptosis analysis in GPMQN-treated cancer cells

**Fig. 3:**
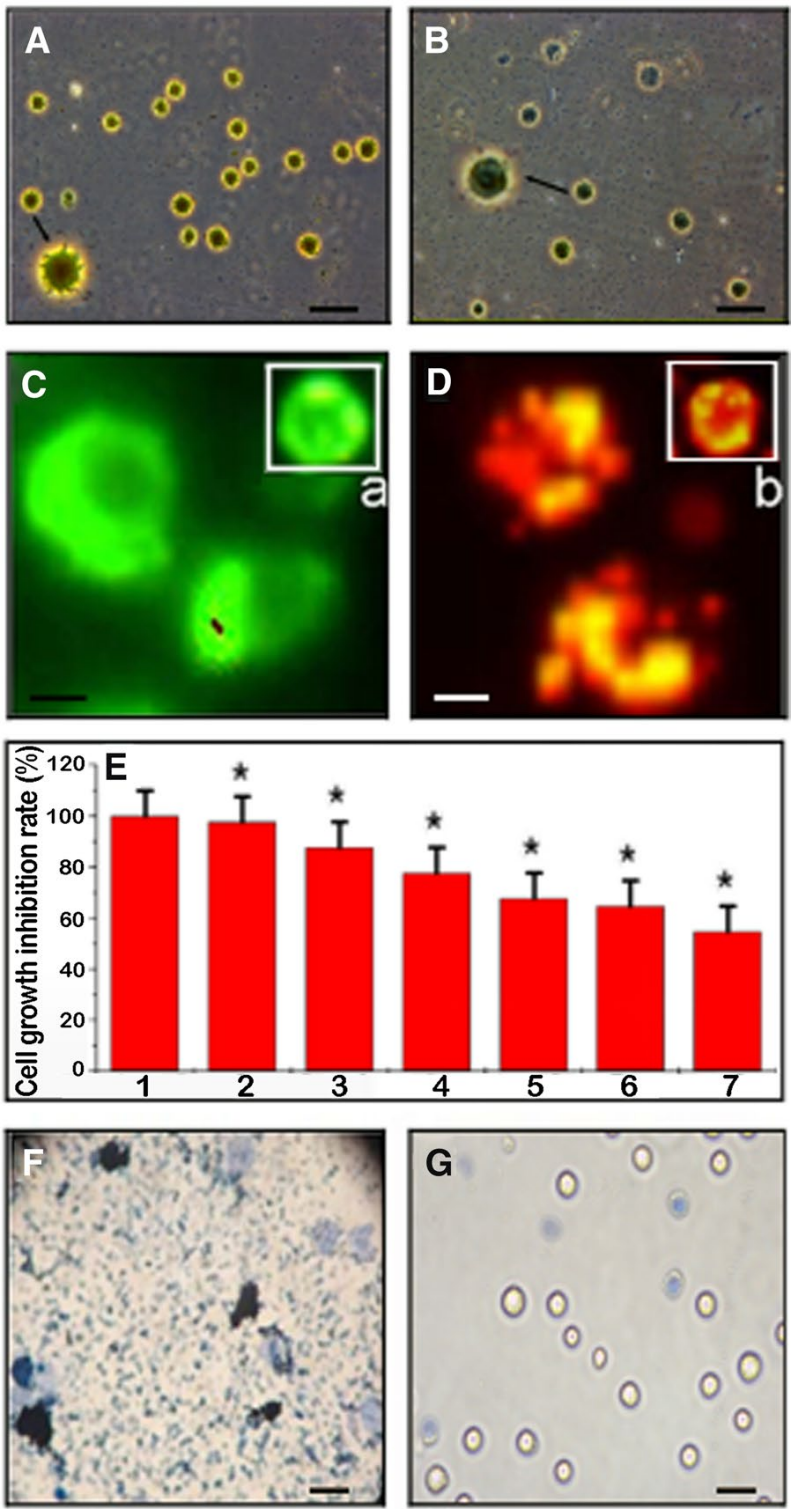
**A** Morphological image of HepG2/ADM cells incubated with 10 mg mL^−1^ GPMQNs (→, amplification image) for 24 h (*Scale bar* = 50 μm); **B** Control. Detection of apoptotic cells by AO/EB Staining, (*Panels* **C**, **D**) apoptotic nuclei from HepG2/ADM cells identified by their distinctively marginated and fragmented appearance. Control cell nuclei cells are observed (*Panels* **C** *a,* **D** *b*) (*Scale bar* 50 μm). **E** Increased growth rate checked by GPMQNs treatments in HepG2/ADM cells. HepG2/ADM cells were treated with *1* 0 mg mL^−1^ GPMQNs (as control), *2* 1 × 10^−4^ mg mL^−1^ GPMQNs, *3* 1 × 10^−3^ mg mL^−1^ GPMQNs, *4* 1 × 10^−2^ mg mL^−1^ GPMQNs, *5* 1 × 10^−1^ mg mL^−1^ GPMQNs, *6* 1 mg mL^−1^ GPMQNs, *7* 10 mg mL^−1^ GPMQNs. Photothermal therapy assay of HepG2/ADM cells treated with GPMQNs. **F** Images of photothermal therapy for cells with 10 mg L^−1^ GPMQNs with laser power threshold of 20 W cm^−2^ for 1 min; **G** Without treatment as control. (*Scale bar* 20 μm), (*P < 0.05 compared to the control group)

The evaluation of normal or apoptotic cells depends on their morphological characteristics. Apoptotic cell membranes (shrinkage, irregular membrane, Fig. 3A) were easily distinguished from normal cell membranes (smooth membrane, Fig. 3B). To further determine the apoptotic effect of GPMQNs in HepG2/ADM cells, an AO/EB staining assay was used. Apoptotic nuclei were identified by their characteristic features such as chromosomal condensation, distinctive margination and fragmentation using fluorescence microscopy. The apoptotic nuclei of HepG2/ADM cells (Fig. 3D, late apoptotic nuclei) treated with GPMQNs for 48 h could be clearly identified by their distinctively red margins and fragmented appearance, compared with the green early apoptotic appearance of cells treated for 24 h (Fig. 3C, early apoptotic nuclei). In the untreated control cells, the cell nuclei were normal, as shown in Fig. 3Ca, Db. Moreover, with increasing GPMQNs concentrations, the growth of the HepG2/ADM cells was strongly suppressed (Fig. 3E, Additional file 1). And the MTT result showed that IC50 value of GPMQNs to HepG2/ADM cells was about the concentration of 1.2 mg mL^−1^.

To further explore the multifunctional anticancer effect of GPMQNs on HepG2/ADM cells, they were probed with a semiconductor laser to perform a hyperthermia experiment. As shown in Fig. 3F, HepG2/ADM cells treated with GPMQNs were severely damaged at a laser power threshold of 20 W cm^−2^ for 1 min. However, no photothermal destruction was observed for HepG2/ADM cell treated with miR-122 at the condition described above (Fig. 3G).

### Molecular mechanisms underlying GPMQNs treatment-induced apoptosis

**Fig. 4:**
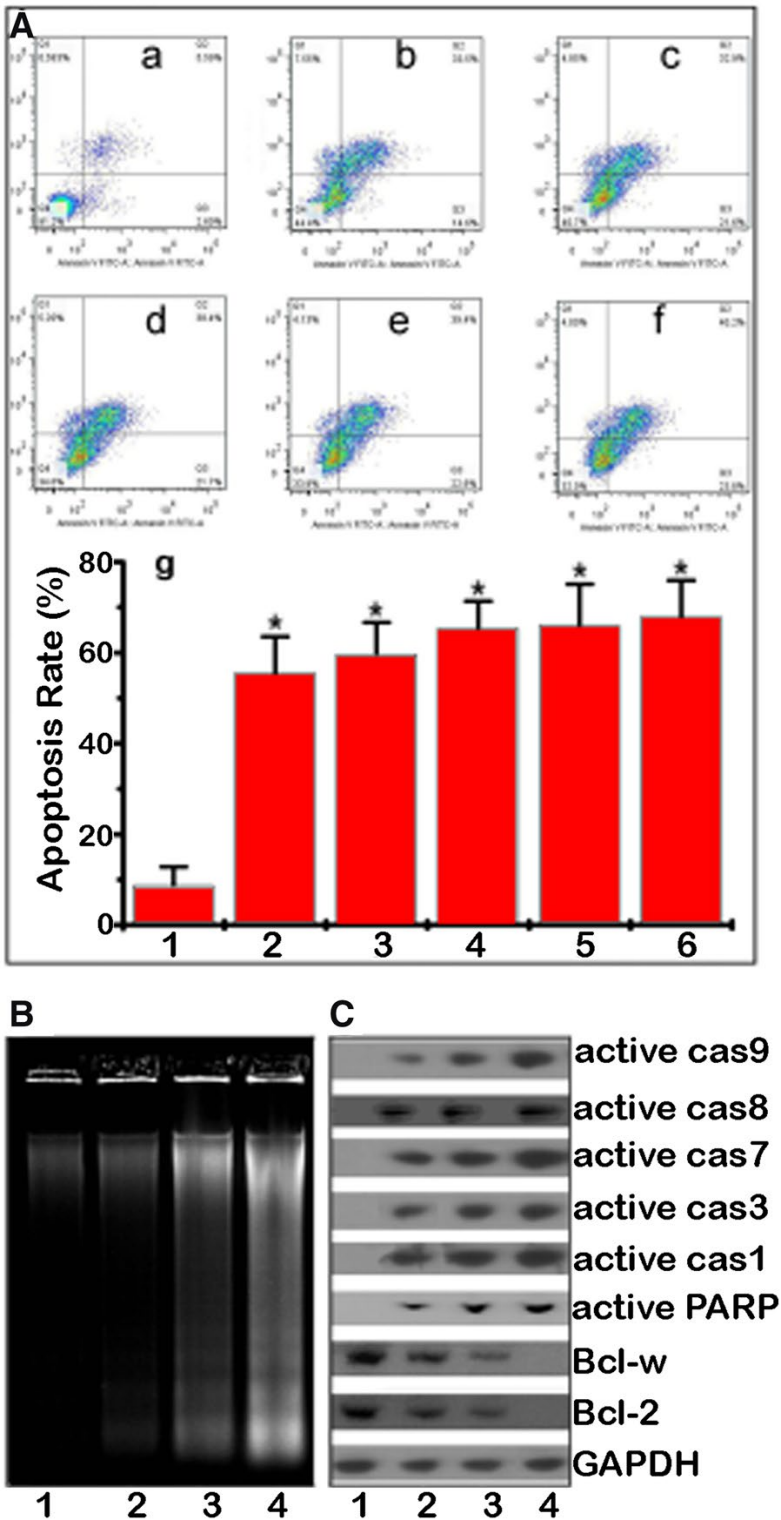
Apoptotic assay of HepG2/ADM cells induced by GPMQNs. **A** Flow cytometric measurement of cellular apoptosis of resistant HepG2/ADM cells treated with various reagents; *a* untreated cells, *b* treatment of cells with 1 mg L^−1^ GPMQNs, *c* treatment of cells with 2 mg L^−1^ GPMQNs, *d* transfected cells with 4 mg L^−1^ GPMQNs, e transfected cells with 8 mg L^−1^ GPMQNs, *f* treatment of cells with 10 mg L^−1^ GPMQNs, *g* histograms of apoptotic rate of HepG2/ADM after various treatments as shown in (*a*, *b*, *c*, *d*, *e*, *f*). **B** DNA fragmentation in resistant HepG2/ADM cells after different treatments; *1* untreated cells, *2* treatment of cells with 10 mg L^−1^ GPMQNs without loading miR-122, *3* transfected cells with miR-122 (1 mg L^−1^, same concentration as loaded on GPMQNs), *4* treatment of cells with 10 mg L^−1^ GPMQNs. **C** Western blot analysis after various treatments; *1* untreated cells, *2* treatment of cells with 10 mg L^−1^ GPMQNs without loading miR-122, *3* transfected cells with miR-122 (1 mg L^−1^, same concentration as loaded on GPMQNs), *4* treatment of cells with 10 mg L^−1^ GPMQNs, (*P < 0.05 compared to the control group)

Using Annexin-V-FITC labeling, the apoptosis induction in treated HepG2/ADM cells was confirmed (Fig. 4A). Significantly, in comparison to the control treatment, the growth inhibition rate was increased when HepG2/ADM cells were treated with gradient concentrations of GPMQNs. Meanwhile, the percentage of apoptotic cells was 68, 66.1, 65.4, 60.3, 55.6, and 8.8% for cells incubated with 10 mg L^−1^ GPMQNs, 8 mg L^−1^ GPMQNs, 4 mg L^−1^ GPMQNs, 2 mg L^−1^ GPMQNs, 1 mg L^−1^ GPMQNs, and control treatment, respectively.

Furthermore, cell apoptosis induced by GPMQNs treatment was confirmed using a DNA fragmentation assay. When HepG2/ADM cells were treated with GPMQNs, the intensity of the fragmented chromosomal DNA bands (Fig. 4B, lane 4) was much higher than that observed in cells treated with miR-122 (Fig. 4B, lane 3) or GPMQNs without miR-122 (Fig. 4B, lane 2). To explore the molecular mechanisms underlying GPMQNs-induced apoptosis, the expression of apoptosis-related proteins in the cells was examined. As shown in Fig. 4C, the protein levels of Bcl-w, which is a target gene of miR-122, was reduced in HepG2/ADM cells after treatment with GPMQNs. Moreover, the cleaved caspase 8 and 9 signals were much stronger in cells treated with GPMQNs (Fig. 4C, lane 4) than in cells treated with GPMQNs without miR-122 or with miR-122 alone (Fig. 4C, lane 2 and 3). The strongest activation of caspases 8 and 9 occurred after GPMQNs treatment (Fig. 4C, lane 4). Similar results were obtained for cleaved caspases 7, 3, and 1 and cleaved PARP, which is a downstream element of the caspase 7, 3, and 1 pathway. The Bcl-2 signal in the HepG2/ADM cells was weaker after GPMQNs treatment than after treatment with GPMQNs without miR-122 or miR-122 alone (Fig. 4C, lanes 4, 3, and 2, respectively). This result suggested that GPMQNs treatment induced the inhibition of antiapoptotic protein activation and caused apoptosis by the activation of caspases 9 and 8 and the Bcl-2 pathway in HepG2/ADM cells.

### Target Tumor Imaging in vivo

**Fig. 5:**
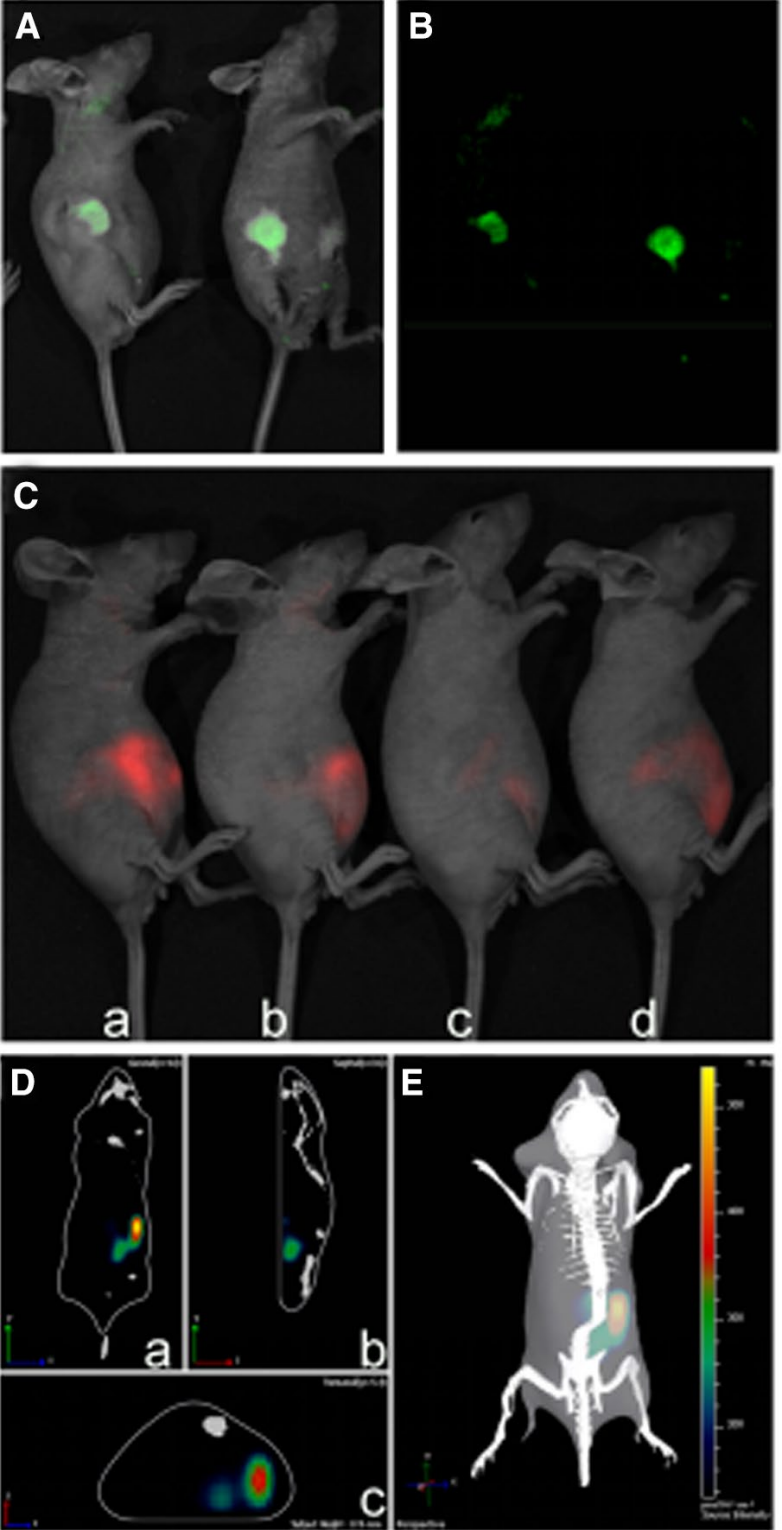
Image of tumor cells in vivo. Fluorescence image of tumor after intravenous injection of 10 mg kg^−1^ GPMQNs (miR-122 with *green fluorescent*) solution for 2 h; **A** fluorescence intensity scan of xenograft tumor, **B** *green fluorescence* near the tumor. **C** Near-infrared fluorescence image of tumor after intravenous injection of 10 mg kg^−1^ GPMQNs solution; *a* treatment for 2 h, *b* treatment for 4 h, *c* treatment for 8 h, *d* treatment for 16 h. **D** 3D reconstruction of HepG2/ADM xenograft tumors in different directions; *a* coronal image, *b* sagittal image, *c* transaxial image. **E** 3D reconstruction of HepG2/ADM xenograft tumors image

Finally, the fluorescence of GPMQNs labeled by different fluorescent dyes was detected in vitro and in vivo. As shown in Fig. 5 A and B, 6-week-old nude mice were subcutaneously implanted with 10^6^ HepG2/ADM tumor cells, which were treated with green fluorescent dye-labeled GPMQNs. Tumors of mice treated with the labeled GPMQNs could produce fluorescence spontaneously. The results illustrated that the intracellular miR-122 content was increased after treatment with GPMQNs in vivo.

The near-infrared fluorescence intensity of tumors after an intravenous injection of 10 mg kg^−1^ of GPMQNs was reduced as the treatment time increased (Fig. 5C). To further test the hypothesis that P-gp antibodies can be used to target drug-resistant tumors, nude mice bearing HepG2/ADM tumors were treated with an intravenous injection of GPMQNs. Near-infrared imaging demonstrated that nude mouse tumors treated with GPMQNs exhibited significant tumor uptake of near-infrared InP QDs in the right lower abdomen of mice from coronal, sagittal, transaxial images (Fig. 5D). 3D reconstruction of nude mice treated with GPMQNs demonstrated that the near-infrared InP QDs fluorescence imaging has been shown to successfully track as fluorescently marked probes. As demonstrated in Fig. 5E, the tumor location could be determined in the HepG2/ADM tumor model clearly and accurately over time by using the 3D representation of GPMQNs.

### Suppression of tumor growth by GPMQNs in nude mice

**Fig. 6:**
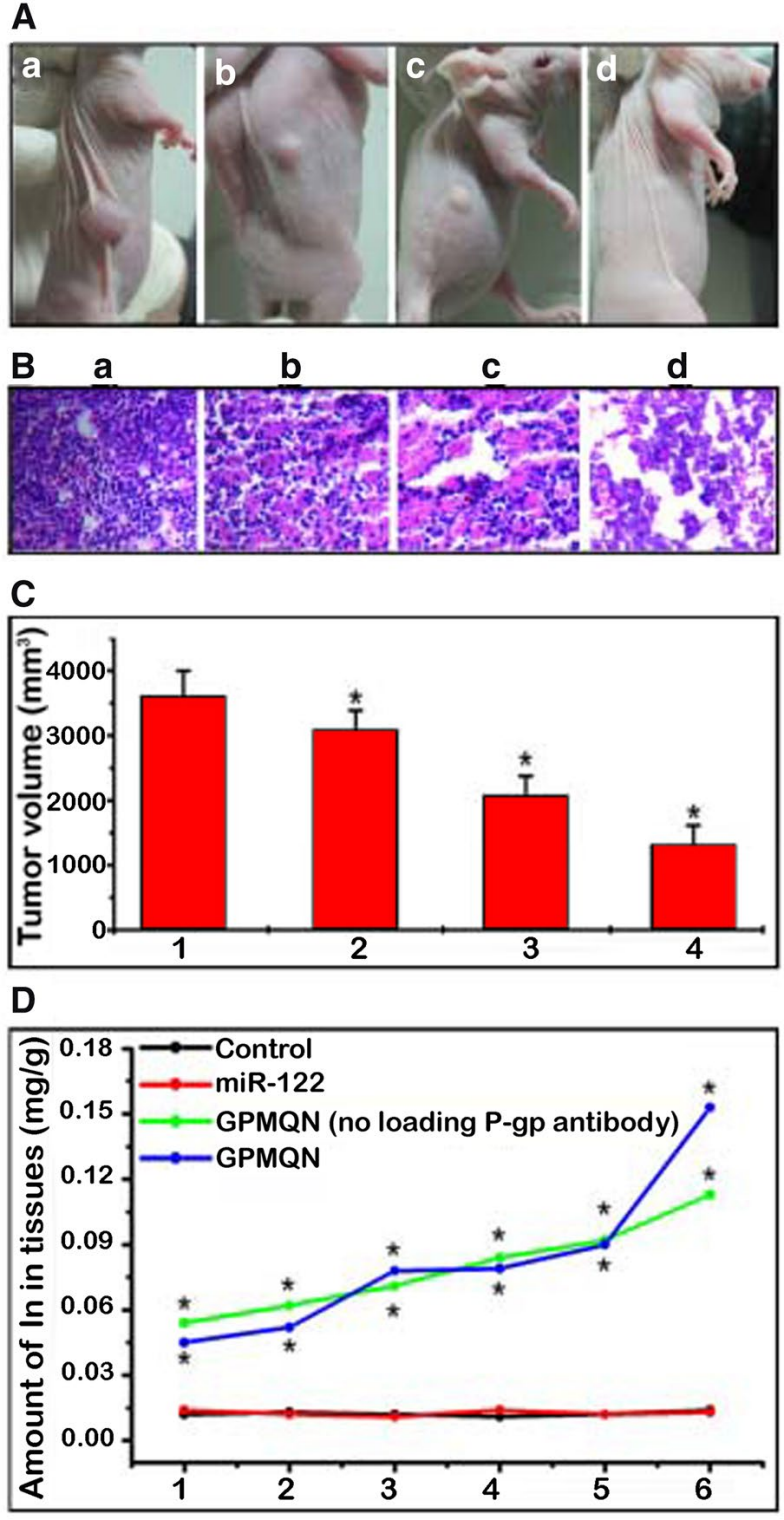
GPMQN therapy assay of HepG2/ADM cells treated with GPMQNs. **A** Inhibition of tumor growth in HepG2/ADM nude mice with different treatments, *a* untreated used as control, *b* treated 10 mg kg^−1^ GPMQNs without loading miR-122, *c* transfected with miR-122 (1 mg kg^−1^, same concentration as loaded on GPMQNs), *d* treated 10 mg kg^−1^ GPMQNs. **B** HE staining was performed on tissue sections of HepG2/ADM xenograft tumors treated as follows: *a* untreated used as control, *b* treated 10 mg kg^−1^ GPMQNs without loading miR-122, *c* transfected with miR-122 (1 mg kg^−1^, same concentration as loaded on GPMQNs), *d* treated 10 mg kg^−1^ GPMQNs. **C** The effect of different treatments on the tumor growth inhibition in HepG2/ADM nude mice. *a* untreated used as control, *b* treated 10 mg kg^−1^ GPMQNs without loading miR-122, *c* transfected with miR-122 (1 mg kg^−1^, same concentration as loaded on GPMQNs), *d* treated 10 mg L^−1^ GPMQNs. **D** Distribution of indium levels in various tissues from different treatment groups. The HepG2/ADM nude mice were treated the same way as shown in (**C**). *1* Brain, *2* Muscle, *3* Skin, *4* Liver, *5* Intestine, *6* Tumor. (*P < 0.05 compared to the control group)

To investigate the effects of GPMQNs on HepG2/ADM tumors in vivo, nude mice were inoculated with HepG2/ADM cells, and the subsequent tumor growth was recorded after various treatments. HepG2/ADM tumor-bearing mice without any treatment exhibited the largest tumor volume (3690 mm^3^, Fig. 6Aa, group 1). The tumor size of the mice treated with GPMQNs (Fig. 6Ad, group 4) was significantly reduced, compared to that of the control group (Fig. 6Aa, group 1) and the groups treated with GPMQNs without miR-122 (Fig. 6Ab, group 2) or miR-122 alone (Fig. 6Ac, group 3).

The synergistic effect of GPMQNs on apoptosis induction in the HepG2/ADM xenograft tumors excised from the nude mice was evaluated (Fig. 6B). As control, the apoptosis rate in the group 1 (untreated HepG2/ADM xenograft tumors, Fig. 6Ba) was approximately 8%. The apoptosis rate in group 2 (treated with GPMQNs without miR-122, Fig. 6Bb) did not increase significantly, was only 23%. However, in group 3 (treated with miR-122 alone, Fig. 6Bc) and group 4 (treated with GPMQNs, Fig. 6Bd), the numbers of apoptotic cells were 34 and 68%, respectively, and were considerably higher in comparison to the control.

To investigate the distribution of GPMQNs in vivo, the tissue uptake of InP QDs was examined. Figure 6D shows the distribution of In levels in various organs. In the mice of group 3 (treated with miR-122 only), the organ distribution of In was the same as that of the control group (Fig. 6D, group 1). However, when GPMQNs were injected into the nude mice of groups 3 and 4, the amount of indium element in all tested organs was higher than the amount in the control group, especially in the tumor, intestines and liver.

## Discussion

As a novel delivery tool, InP QDs (positively charged) could be easily fixed non-coding RNAs miR-122 (negatively charged). InP QDs and miR-122 are amphiphilic substances (both positive and negative electrical attraction) that form the core–shell structure of the miR-122-containing nanocarriers. Bao et al. [22] have developed chitosan functionalized graphene oxide as a nanocarrier for drug and gene delivery. InP QDs-miR-122 formed a nanocomposite that was further adhered onto grapheme oxide. In reality, the size of the graphene oxide is a problem that affects its use as a drug carrier. If the graphene oxide sizes are too large, drug delivery in the blood stream would be affected. Whereas if graphene oxide sizes are too small, drug loading would be affected, and particle phagocytosis would readily occur [23]. Thus, in the present study, we concluded that the size of graphene oxide should be approximately 300 nm (achieved through gradient centrifugation) to ensure its drug loading and to avoid phagocytosis by phagocytic cells in vivo. As mentioned above, this size range could make use of nano-drug biodistribution through the enhanced permeability and retention effect to target tumors.

GSH was able to control the miR-122 release from the GPMQNs by ligand displacement, so we could achieve sustained controlled release of miR-122 in cancer cells. The concentration of GSH in erythrocytes was 2 mM, whereas it was 10 mM in the HepG2/ADM cells. Due to the P-gp antibody, the GPMQNs could target to the HepG2/ADM cell membrane. In this study, cancer cells were targeted via antigen–antibody interactions. As described above, InP QDs loaded with miR-122 were formed as nanocomposites, and the nanocomposite was adhered onto graphene oxide to induce the apoptosis of drug-resistant cancer cells (InP QDs incorporated onto the graphene oxide with chitosan functionalization). As the predominant low-molecular-weight thiol in animal cells, GSH provided a potential environment for miR-122 entry into HepG2/ADM cells [24]. Besides its pleiotropic metabolic effects, miR-122 was selected instead of chemical drugs for another two reasons: to avoid the high toxicity of chemical drugs, and attempt to avoid multi-drug resistance to chemotherapy [25].

HepG2/ADM membrane permeability and intracellular miR-122 accumulation assays were performed. After GPMQNs treatment, an increase in the amount of intracellular miR-122 was observed in the HepG2/ADM cells. It demonstrated that cell membrane permeability was significantly increased by GPMQNs treatment, which induced the uptake of miR-122 in HepG2/ADM cells. These results suggested that the InP QDs could be readily internalized by cells for drug delivery.

Photothermal activity can deposit a sufficient amount of energy into tissue under appropriate conditions to raise the temperature above a certain threshold so that cancer cell destruction would occur. Multifunctional GPMQNs have been proven to effectively enhance the photothermal killing of tumor cells by lasers in vitro. As shown in the results, cancer cells treated with GPMQNs were destroyed at the laser power threshold of 20 W cm^−2^ for 1 min, and the tumor growth was suppressed. This result was indeed consistent with earlier observations of laser treatments for photothermal therapy in vitro [26].

Next, the apoptotic effects of GPMQNs on HepG2/ADM cells were further detected by MTT assay, nuclear staining, and a DNA fragment assay. The GPMQNs were demonstrated to exhibit an anti-proliferative effect in a dose-dependent manner in HepG2/ADM cells. Using AO/EB staining of apoptotic cells, apoptotic nuclei were identified by their distinctively marginated and fragmented appearance. The apoptotic nuclei of HepG2/ADM cells at 48 h could be identified by their distinctively marginated and fragmented appearance. In the control cells without treatment, the cell nuclei were normal. The apoptosis effects on the cell nuclei allow DNA extraction methods to generate apoptosis DNA ladders in apoptotic cells. To determine whether cell growth inhibition was caused by the apoptotic response, we showed that the relevant GPMQNs induced a much higher cell apoptosis rate than the untreated control using an Annexin-V-FITC and PI apoptosis detection method. The apoptotic DNA ladders were examined by agarose gel electrophoresis. In HepG2/ADM cells treated with GPMQNs, the intensity of the apoptosis DNA ladders was much higher than that observed in untreated cells or cells treated with miR-122 alone. Our observations supported the hypothesis that the remarkable enhancement of apoptosis was induced by the synergistic effect of the GPMQNs. Anticancer research on the GPMQNs apoptosis signaling pathway was carried out as well. According to previous research reports, miR-122 could induce apoptosis through the Bcl-w pathway involving hepatoma therapy [27, 28]. However, the underlying molecular mechanisms of the induction of cancer cell apoptosis by graphene-InP QDs material and miR-122 remain unclear. This study highlighted the mechanism of apoptosis induction in drug-resistant HepG2/ADM cells treated by GPMQNs. We found that GPMQNs treatment activated the Bcl-w and caspase 8 and 9 pathways to induce apoptosis in HepG2/ADM cells. The cleaved caspase 8 and 9 activated caspases 3, 7, and 1, which correlated with the increased cleaved PARP expression after GPMQNs treatment. Apoptotic DNA ladders were induced during cell apoptosis by the expression of cleaved PARP.

Over the past two decades, cadmium chalcogenide QDs such as cadmium sulfide (CdS), cadmium selenide (CdSe), and cadmium telluride (CdTe) QDs have been extensively studied [29]. In particular, CdSe/ZnS (core/shell) QDs have been well-studied in terms of synthesis and its biological applications [30]. Because of the potential toxicity concerns for heavy metal ions such as Cd^2+^, other alternative QDs that do not contain heavy metal elements have been investigated, including InP/ZnS (core/shell) QDs. Visible fluorescence penetration capability is very poor, and imaging with the above-mentioned QDs cannot be achieved in vivo. For bioimaging applications that demand deep tissue penetration, near-infrared fluorescence is more advantageous for QD emission because it provides greater tissue penetration due to reduced photon scattering and auto-fluorescence. Many near-infrared emitting QDs, including lead selenide (PbSe), CdTe/CdSe (core/shell), and lead sulfide (PbS), have been developed [31, 32]. These QDs also encounter the problem on high toxicity of heavy metal, however, the toxicity of InP QDs is relatively low. With tumor bioimaging by fluorescent, the GPMQNs were found to enhance the imaging of HepG2/ADM tumor features in vivo. Such result was expected to improve the consistency of target volume measurements for treatment. The image results offer an advantage for applications that demand deep tissue penetration target activity.

Meanwhile, the potential tumor inhibition effect of the GPMQNs, which affected cellular metabolism and increased the apoptosis of tumor cells, was explored in vivo. The anti-cancer effect of the GPMQNs was evaluated by investigating the extent of apoptosis induction by HE staining in vivo. In agreement with the in vitro signal expression results, the HepG2/ADM tumors exhibited considerable induction of apoptosis-related proteins in mice treated with GPMQNs. Compared to the other groups, the indium concentration in the nude mice injected with GPMQNs was significantly higher in tumor tissues, and this phenomenon illustrated their targeting property. The following distribution tissues of indium were intestines and liver, and it was proposed that GPMQNs may be subsequently excreted via intestines or liver, which warranted further investigation.

## Conclusions

In conclusion, our experimental results indicated that the delivery of miR-122 by the multifunctional GPMQNs would provide a novel and effective system to induce apoptosis and inhibit the growth of hepatic tumor cells. And herein, GPMQNs were demonstrated to possess the following advantages: (1) low toxicity, (2) selective targeting, (3) intracellular drug accumulation enhancement, (4) apoptosis promotion of drug-resistant liver cancer cell, (5) the near-infrared imaging. Therefore, GPMQNs may be a promising nano-strategy for treating drug-resistant hepatoma cells, which suggested that it warrants further evaluation as cancer therapeutics in clinical.

## Additional file

**Additional file 1.** Increased growth rate checked by GPMQNs treatments in HepG2/ADM cells via Cell Counting Kit-8 (CCK-8) assay. The results were consistent with MTT assay, and demonstrated no assay interference caused by the interactions between nanoparticles and assay substrates leading to false-positive/ false-negative results.

## Abbreviations

MDR: multi-drug resistance
P-gp: P-glycoprotein
GST: glutathione transferase
MRI: magnetic resonance imaging
QDs: quantum dots
InP: indium phosphide
ZnS: zinc sulfide
miRNAs: microRNAs
RGO: reduced graphene oxide
GPMQNs: graphene-P-gp loaded with miR-122-InP@ZnS quantum dots nanocomposites
GSH: glutathione
NaCl: sodium chloride
H_2_SO_4_: sulfuric acid
KMnO_4_: potassium permanganate
H_2_O: water
H_2_O_2_: hydrogen peroxide
HCl: hydrochloric acid
ddH_2_O: double-distilled water
TEM: transmission electron microscope
NaOH: sodium hydroxide
EDC: 1-(3-dimethylaminopropyl)-3-ethylcarbodiimide
ECL: enhanced chemiluminescence
In(AC)_3_: indium acetate
MA: myristic acid
ODE: 1-octadecene
HAc: acetic acid
CCD: charge coupled device
FCS: fetal calf serum
CO_2_: carbon dioxide
LDH: lactate dehydrogenase
AO/EB: acridine orange/ethidium bromide
MTT: 3-(4,5-dimethylthiazol-2-yl)-2,5-diphenyltetrazolium bromide
DMSO: dimethyl sulfoxide
PARP: proteolytic cleavage of poly-(ADP-ribose) polymerase
GAPDH: glyceraldehyde-3-phosphate dehydrogenase
HE: hematoxylin-eosin
3D: three-dimensional
CdS: cadmium sulfide
CdSe: cadmium selenide
CdTe: cadmium telluride
PbSe: lead selenide
PbS: lead sulfide
