# Supplementary material for: Targeted imaging and induction of apoptosis of drug-resistant hepatoma cells by miR-122-loaded graphene-InP nanocompounds
Source: J Nanobiotechnology. 2017 Jan 23;15:9. doi: 10.1186/s12951-016-0237-2 (PMC5260086; doi:10.1186/s12951-016-0237-2)
Supplement: Supplementary file 1 — Additional file 1. Increased growth rate checked by GPMQNs treatments in HepG2/ADM cells via Cell Counting Kit-8 (CCK-8) assay. The results were consistent with MTT assay, and demonstrated no assay interference caused by the interactions between nanoparticles and assay substrates leading to false-positive/ false-negative results. [file 12951_2016_237_MOESM1_ESM.doc]

**Additional file 1.**

HepG2/ADM cells were plated in 96-well plates (2×10^3^ cells/well). After overnight incubation, the cells were treated with various concentrations of GPMQNs. After 36 hours, 10 μL Cell Counting Kit-8 (CCK-8) (Dojindo Laboratories, Kumamoto, Japan) was added into each well. Absorbance was measured at 450 nm using a microplate reader.

Additional figure 1. Increased growth rate checked by GPMQNs treatments in HepG2/ADM cells. HepG2/ADM cells were treated with 1: 0 mg mL^-1^ GPMQNs (as control), 2: 1×10^−4^ mg mL^-1^ GPMQNs, 3: 1×10^−3^ mg mL^-1^ GPMQNs, 4: 1×10^−2^ mg mL^-1^ GPMQNs, 5: 1×10^−1^ mg mL^-1^ GPMQNs, 6: 1 mg mL^-1^ GPMQNs, 7: 10 mg mL^-1^ GPMQNs.
